# Supplementary material for: Monomeric IgA Antagonizes IgG-Mediated Enhancement of DENV Infection
Source: Front Immunol. 2021 Nov 24;12:777672. doi: 10.3389/fimmu.2021.777672 (PMC8654368; doi:10.3389/fimmu.2021.777672)
Supplement: Supplementary file 1 [file DataSheet_1.pdf]

Supplemental Figure 1: Neutralization assay gating strategy

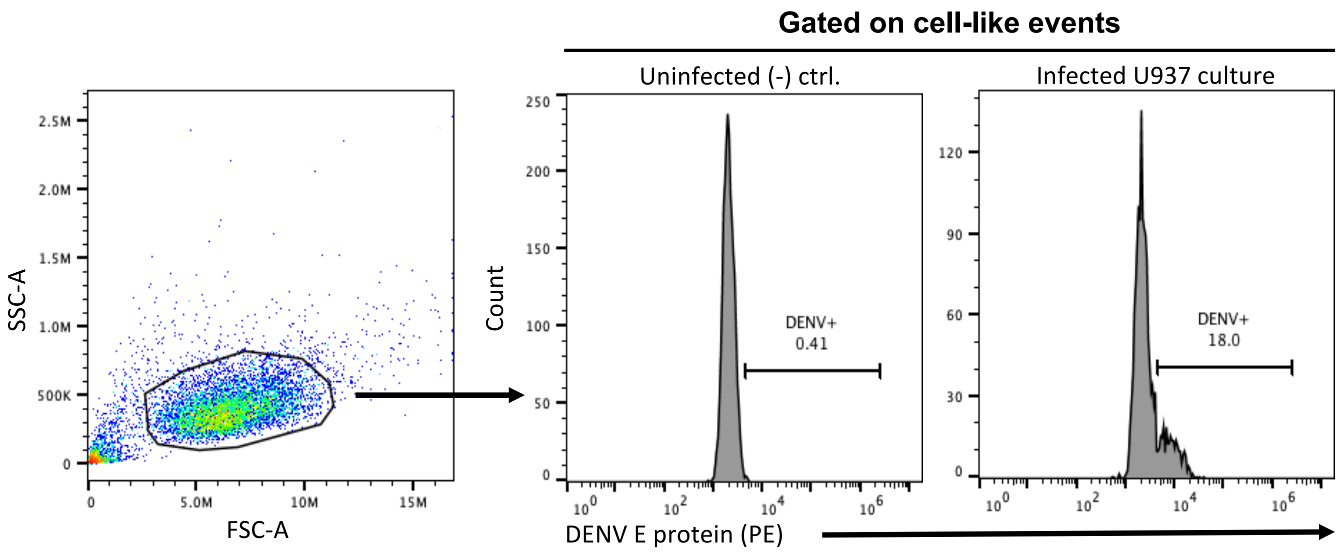

Supplemental Figure 1. Gating scheme and **r**epresentative plots from FlowNT assays

Supplemental Figure 2: ADE assay gating strategy

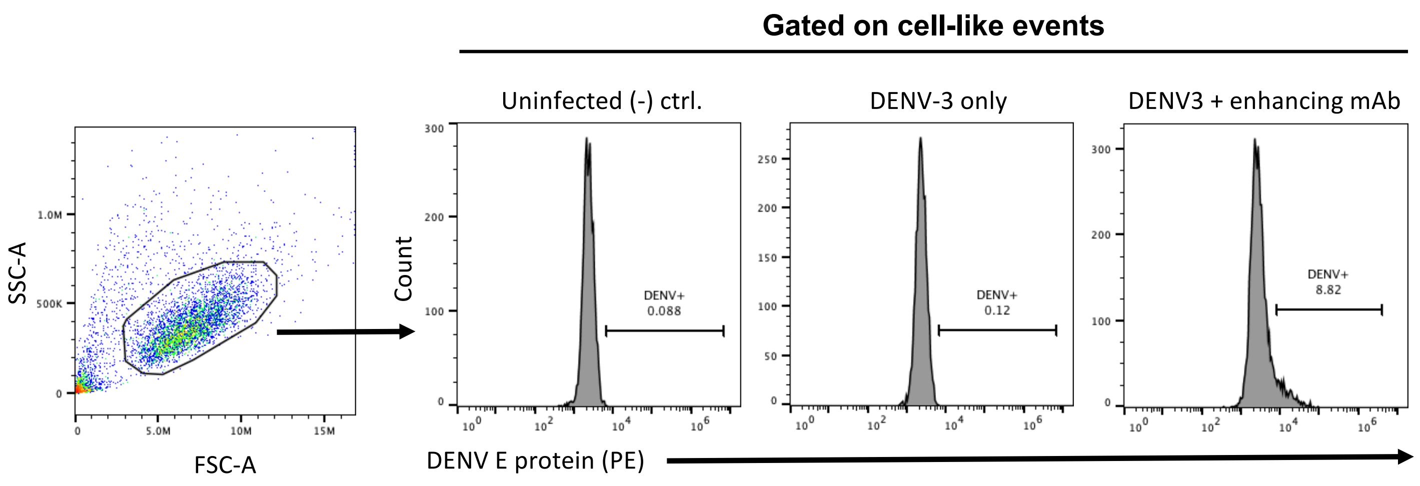

Supplemental Figure 2. Gating scheme and **r**epresentative plots from ADE assays

Supplemental Figure 3: ELISA for IgM/IgG/IgA in DENV-immune plasma samples

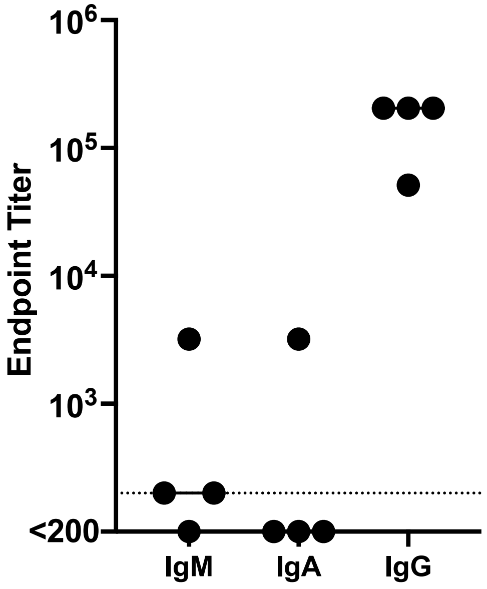

Supplemental Figure 3. DENV-3 IgM, IgG, and IgA titers in DENV-immune plasma samples utilized in ADE assay

Supplemental Figure 4: DENV neutralization and ADE capacity of DENV-immune plasma samples

A)

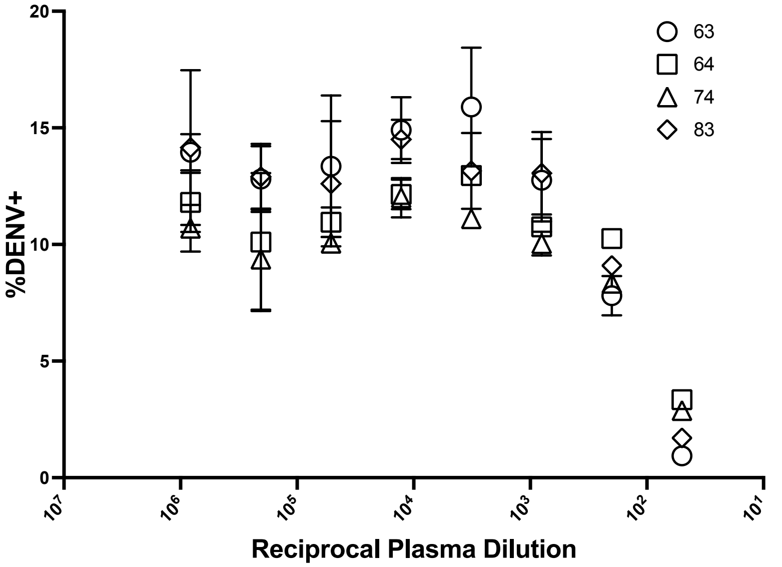

B)

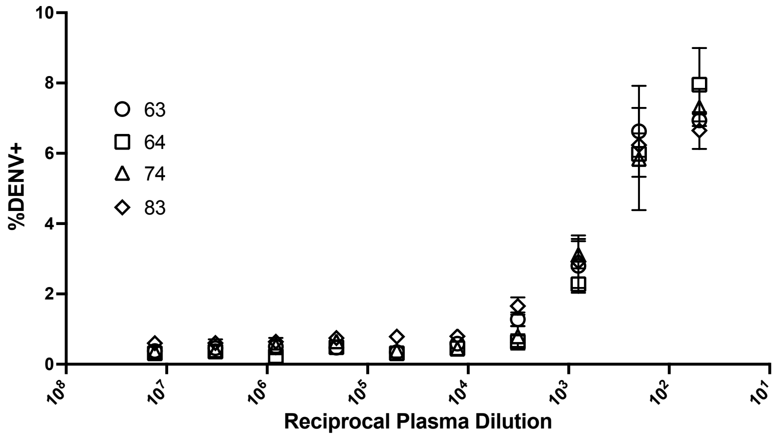

Supplemental Figure 4. A) DENV-3 neutralization activity of DENV-immune plasma as assessed by FlowNT. **B)** DENV-3 ADE activity of DENV-immune plasma as assessed by K562 infection. Error bars +/- SEM

Supplemental Figure 5: Nonspecific IgA does not suppress DENV ADE *in vitro*

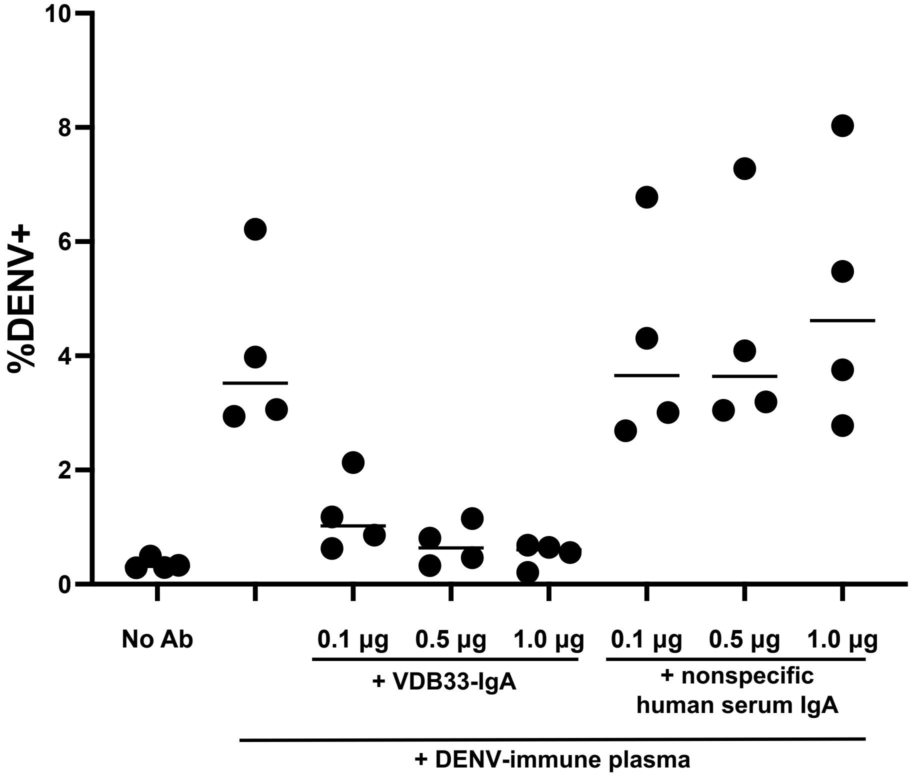

Supplemental Figure 5: Nonspecific human IgA does not antagonize polyclonal plasma-mediated antibody-dependent enhancement.
