# Supplementary material for: Monomeric IgA Antagonizes IgG-Mediated Enhancement of DENV Infection
Source: Front Immunol. 2021 Nov 24;12:777672. doi: 10.3389/fimmu.2021.777672 (PMC8654368; doi:10.3389/fimmu.2021.777672)
Supplement: Supplementary file 2 [file Table_1.docx]

**Supplemental Table 1. Dengue immune plasma used in this study**

| **Supplier** | **Product #** | **Donor ID** | **Batch number** |
| --- | --- | --- | --- |
| SeraCare | 0325-0014 | BD250524 | 10127363 |
| SeraCare | 0325-0014 | BD250525 | 10127364 |
| SeraCare | 0325-0014 | BD250535 | 10127374 |
| SeraCare | 0325-0014 | BD250543 | 10127383 |
